# Cohesive Zone Modeling of the Interface Fracture in Full-Thermoplastic Hybrid Composites for Lightweight Application

**DOI:** 10.3390/polym15224459

**Published:** 2023-11-19

**Authors:** Ruggero Giusti, Giovanni Lucchetta

**Affiliations:** Department of Industrial Engineering, University of Padua, Via Venezia 1, 35131 Padova, Italy

**Keywords:** cohesive zone modeling, fracture, injection-molding

## Abstract

With the increasing demand for lightweight and high-performance materials in the automotive and aerospace industries, full-thermoplastic hybrid composites have emerged as a pivotal solution, offering enhanced mechanical properties and design flexibility. This work aims to numerically model the fracture strength in full-thermoplastic hybrid composites made by forming and overmolding organosheets. The mode I fracture was investigated by modeling the behavior of T-joint specimens under a tensile test following the cohesive zone modeling (CZM) approach. The sample was designed to replicate the connection between the laminate and the overmolded part. Double cantilever beam (DCB) specimens were manufactured with organosheets and tested to mode I opening to determine the interlaminar fracture toughness. The fracture toughness out of the mode I test with DCB specimens was used to define the CZM parameters that describe the traction-separation law. Later, due to the particular geometry of the T-join specimens that under tensile load work close to pure mode I, the cohesive parameters were determined by inverse analysis, i.e., calibrating the theoretical models to match experimental results. The fracture resistance T-joint specimens appeared dependent on the fiber-bridging phenomenon during the delamination. In particular, the presence of fiber-bridging visible from the experimental results has been replicated by virtual analyses, and it is observed that it leads to a higher energy value before the interface’s complete breakage. Moreover, a correspondence between the mode I fracture toughness of the DCB specimen and T-joint specimens was observed.

## 1. Introduction

Hybrid composite materials are increasingly used in the automotive industry to answer to the lightweight requirements of the regional transportation regulations that require reducing the vehicle weight to reduce energy consumption and local CO_2_ emissions [1,2,3,4]. Plastic composites are the best candidates for reducing automotive parts’ weight because of their high specific performance. Moreover, they can be combined with other materials in a more complex final part through junctures based on different adhesion mechanisms [5].

When hybridization exploits the thermoplastic composites over the thermosetting ones, additional benefits can arise with metal-replacement operations to fulfill the end-of-life vehicle directive, promoting recycling and reuse maximization [6]. Moreover, the higher processability and the lower cycle time of thermoplastic composites lead to lower energy consumption for the manufacturing process [7].

Recent investigations have highlighted the need for a deeper understanding of the behavior of overmolded short/continuous-fiber-reinforced thermoplastic composites under extreme conditions, including their moisture absorption characteristics and consequent mechanical behavior [8]. In the last decade, many technologies have taken advantage of the design freedom that thermoplastic materials offer by exploiting their recyclability and the versatility of the injection-molding process. The main advantages are the suitability for high-volume production and the implementation of the in-mold assembly or joining [9,10]. For instance, in the insert-molding process, a pre-treated insert is fixed inside the mold before the injection phase [11]. The in-mold bonding integrates dispensing an adhesive on the mold insert before the injection phase [12,13]. The in-mold forming technology directly couples the thermoforming of so-called organo-sheets with the injection-molding, and it takes advantage of the compatibility of the polymer matrix that heals the interface when they merge at their molten temperature [14,15]. The role of interface temperature in determining the mechanical properties, including the low-velocity impact response of short/continuous-fiber-reinforced composites, has been demonstrated to be significant [16].

In particular, in-mold forming technology (IMF), which is also known as FiberForm, Organomelt, and Spriform, was proposed for the manufacturing of full-thermoplastic hybrid composites where an outer thermoformed shell reinforced with continuous fibers is internally stiffened with an over-injected ribs system [14]. 

Due to the high investment in tooling, finite element simulations are essential to design the part and the mold correctly. Currently, the main difficulty in modeling is defining the welding strength between parts [15]. In the specific case of full-thermoplastic composites, the theoretical strength can equal the matrix material [17]. Even if good welding occurs, the weaker zone moves to the laminate side at the interface between the matrix and the glass-woven reinforcement, and the direct consequence is a weaker zone at the welding area that could be subjected to delamination failure [18,19,20,21].

Overmolded composites, combining continuous- and short-fiber reinforcements, have shown superior structural performance and lightweight advantages, making them the materials of choice for complex load-bearing structures [22]. From the numerical point of view, delamination can be commonly simulated with the cohesive zone models that were initially proposed by Barrenblatt and Dugdale [23,24] and that, over the years, have been reformulated and adapted to many different cases [25,26,27,28,29]. Cohesive laws are used to model the fracture in various applications, which include metal–ceramic interfaces [30], the use of adhesive in the bonding of metals and composites [31,32,33,34,35,36], and overmolded composites [37]. The influence of preheating processes on the interfacial bonding and subsequent mechanical behaviors such as tensile, flexural, and interlaminar fracture toughness in overmolded composites has been proven to be substantial, underscoring the importance of detailed interfacial analysis in cohesive zone modeling [38].

In this study, an investigation is conducted into the fracture behavior of full-thermoplastic hybrid composites, focusing on the interface fracture in hybrid T-joint specimens. This work introduces the use of cohesive zone modeling (CZM) to predict the behavior of these composites, an approach not extensively explored in previous research, particularly considering the specific geometries and materials used in this study.

The methodology combines experimental and computational methods. Double cantilever beam (DCB) tests were conducted to ascertain the interlaminar fracture toughness of the material, informing the parameters for the CZM. This method is essential for simulating fracture propagation, supporting the hypothesis that fracture behavior in these composites can be accurately predicted with CZM.

The paper is structured to sequentially present the research process and findings. It starts with the description of the DCB specimen preparation and testing, followed by an explanation of the application of CZM in simulating fracture behavior. Results from both experimental and computational analyses are then presented, leading to a discussion that places these findings within the existing body of knowledge. The paper concludes with a reflection on the study’s implications and suggestions for further research in the field.

## 2. Theoretical Background

The concept of the CZM method considers the fracture mechanism as a gradual phenomenon during which the separation between the surfaces develops in a narrow region at the crack tip, and forces of cohesive nature counteract it. The cohesive law, better known as traction-separation law, governs the relationship between these forces and the separation of the surfaces near the apex of the crack.

Barenblatt [25] formulated the cohesive zone modeling for the first time as an alternative to fracture mechanics in a perfectly brittle material, while Dugdale [26] extended the concept to perfectly plastic materials. A formulation of cohesive laws must be selected to describe the crack tip propagation. Some of the most known formulations include exponential [27], polynomial [28], trapezoidal [29], as well as bilinear [30] and trilinear [31]. Each of the laws has an initial portion in which the cohesive stress (cohesive traction) is growing and a separation level in which the value of the tension is maximum and then is followed by a decreasing trend, indicating the damage of the material and the decay of its strength. 

When the traction value becomes zero, the material can no longer offer any resistance. The area under the traction-separation curve expresses the work of separation per unit area accomplished for opening the two virtual surfaces. This quantity is linked to the fracture toughness of the material or of the interface. In the case of pure opening, the area under the curve (normal traction-separation law) represents the critical energy release rate due to mode I opening ($G_{Ic}$). $G_{Ic}$ represents the value of the strain energy release rate that leads to the complete failure or damage of the element and, as a consequence, to the propagation of the crack. In this work, bi-linear and the tri-linear laws are used to describe the behavior of the tested specimens. 

### 2.1. Bilinear Law

**Figure 1:**
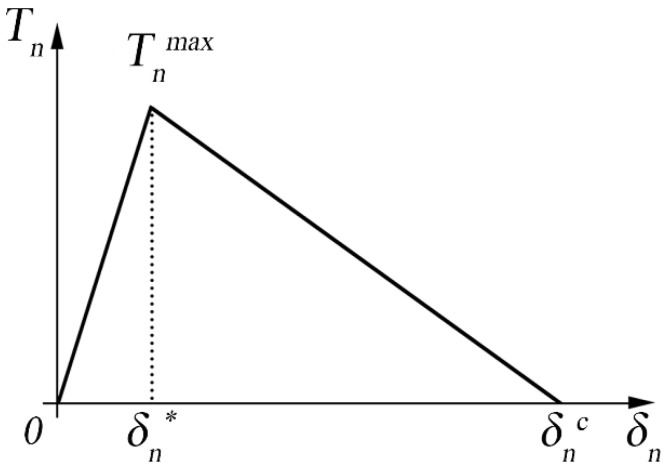
Graphical representation of the bilinear cohesive law.

Geubelle and Baylor [30] initially proposed the bilinear law (or triangular law) to describe the delamination in composites by assuming that it refers to the pure mode I opening. Figure 1 indicates the graphical representation of the bi-linear law, where the area under the curve is proportional to the critical energy that an element can reach before the propagation of the crack, as described by Equation (1).

**Figure 2:**
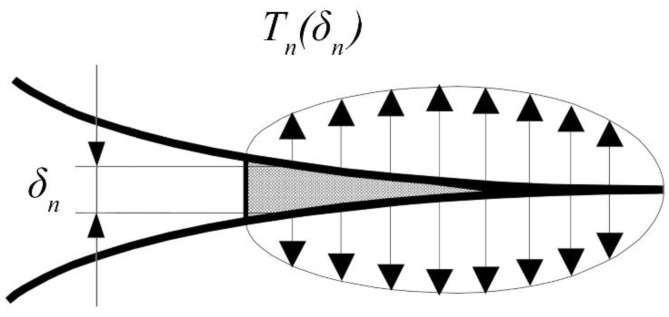
Loading condition at the crack tip.

The behavior is linear and elastic between $\delta_{n}=0$ and $\delta_{n}=\delta_{n}^{*}$, where there is the total reversibility of the cohesive zone. The area under the curve until $\delta_{n}^{c}$ defines the critical energy required before obtaining the propagation of the crack.

(1) $$G_{IC}=\frac{1}{2}\;T_{n}^{max}\;\delta_{n}^{c},$$

where $T_{n}^{max}$ indicates the maximum cohesive stress at the interface, while $\delta_{n}^{c}$ indicates the displacement at the crack tip. Figure 2 shows a typical loading condition at the crack tip. If the displacement is between 0 and $\delta_{n}^{*}$, the element is going to increase the resistance offered against the opening. After $\delta_{n}^{*}$, the resistance of the element is going to decrease until the complete failure of the element when displacement $\delta_{n}^{c}$ is reached, and the element does not participate anymore in providing resistance.

In other words, at $\delta_{n}^{*}$ the damage of the interface starts in an un-reversible way, and the critical value is reached for $\delta_{n}=\delta_{n}^{c}$, which corresponds to the total damage of the interface. The area under the traction-separation curve expresses the unitary work of the separation that represents the strain energy release rate $G_{I}$. The formulation of the bilinear law is uniquely defined after defining the rate α in Equation (2).

(2) $$\alpha =\frac{\delta_{n}^{*}}{\delta_{n}^{c}},$$

The model assumes that the fracture is dominated by the normal separation of the interface. 

### 2.2. Trilinear Law

**Figure 3:**
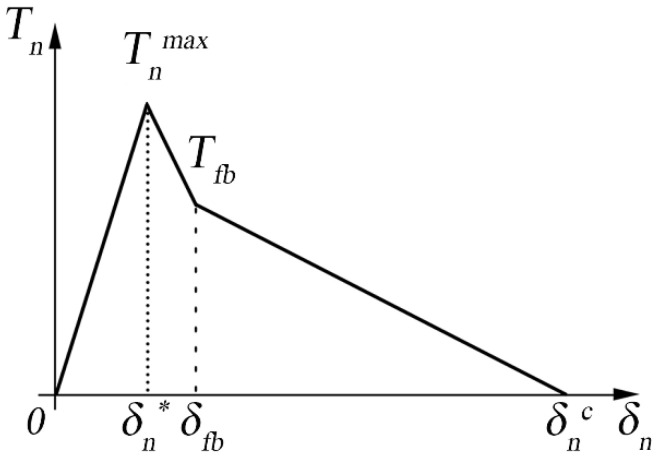
Graphical representation of the trilinear cohesive law.

The trilinear cohesive law was subsequently introduced, and it has proved particularly suitable to describe the behavior of polymeric materials with reinforcing fibers. The trilinear law was used when the crack front propagation was accompanied by a significant leakage of the fibers from the matrix [31]. This phenomenon is generally indicated as fiber-bridging. From a simplistic point of view, the trilinear cohesive law can be seen as the sum of two bilinear laws that are merged to form a single law, as represented in Figure 3, where the first is associated with the breaking of the matrix, whereas the latter represents the fiber-bridging. 

The second part of a trilinear cohesive law is particularly suitable for describing the fiber-bridging phenomenon in composite materials due to its ability to model the material behavior during the damage evolution phase. In CZM, the trilinear law typically consists of three distinct phases:

- Initial linear-elastic phase: Represents the initial stiffness of the interface up to the onset of damage.
- Damage evolution phase (softening phase): This is where the second part of the trilinear law becomes crucial. After the peak load is reached and initial damage occurs, the material does not fail immediately but exhibits a softening behavior, where the load-bearing capacity gradually decreases with increasing separation. This phase can effectively model the fiber-bridging phenomenon. Fiber-bridging occurs when fibers span the crack faces, maintaining some load transfer across the crack even after initial failure. This phase in the trilinear law can represent the gradual loss of stiffness and strength due to the progressive failure of these bridging fibers.
- Final failure phase: This part represents the final part of the curve where the material completely fails and can no longer bear any load.

In addition to the bi-linear law, there are two additional parameters: $T_{n}^{fb}$ and $\delta_{n}^{fb}$, which identify the phenomenon of fiber-bridging. The point in the correspondence of the slope change at the damaging front identifies these parameters. Working in displacement control, the reference energy quantity is the strain energy release rate. The critical value corresponding to crack propagation will provide the mode I interlaminar fracture toughness, $G_{Ic}$.

The law is defined by the characteristic values of the displacement and the value of the parameter $\alpha_{\sigma }$, shown in Equation (3), which represents the rate between the maximum cohesive stress $T_{n}^{max}$ and the cohesive stress at the beginning of the fiber-bridging $T_{n}^{fb}$.

(3) $$\alpha_{\sigma }=\frac{T_{n}^{fb}}{T_{n}^{max}},$$

The ASTM D5528 standard [39] was followed for the creation of the double cantilever beam (DCB) to perform the mode I test and to calculate the strain energy release rate $G_{I}$ for the organosheet material. The $G_{I}$ values were calculated by averaging the three methods defined by the standard, i.e., the Modified Beam Theory (MBT), the Compliance Calibration (CC) method, and the Modified Compliance Calibration (MCC) method.

In the Modified Beam Theory (MBT), the strain energy release rate is expressed as:

(4) $$G_{MBT}=\frac{3\;P\;u}{2\;b\;a},$$

In Equation (4), *P* represents the applied load, *u* is the load-line displacement, *b* is the specimen width, and *a* is the crack length.

In the Compliance Calibration (CC) Method, $G_{I}$ is calculated using Equation (5):

(5) $$G_{CC}=\frac{P^{2}}{2b}\frac{\partial C}{\partial a},$$

Here, *P* is the applied load, *b* is the specimen width, *C* stands for the compliance (*C* = *u*/*P*), and ∂C/∂a is the derivative of compliance with respect to the crack length.

The Modified Compliance Calibration (MCC) method uses the following expression to determine $G_{I}$:

(6) $$G_{MCC}=\frac{{\pi aP}^{2}}{bE^{\prime }h^{3}},$$

In this equation, *P* is the applied load, *a* is the crack length, *b* is the specimen width, *E′* is the effective modulus of the beam accounting for shear deformation and rotational effects at the crack tip, and *h* is the thickness of the specimen.

## 3. Experimental Section

### 3.1. DCB Specimens

**Figure 4:**
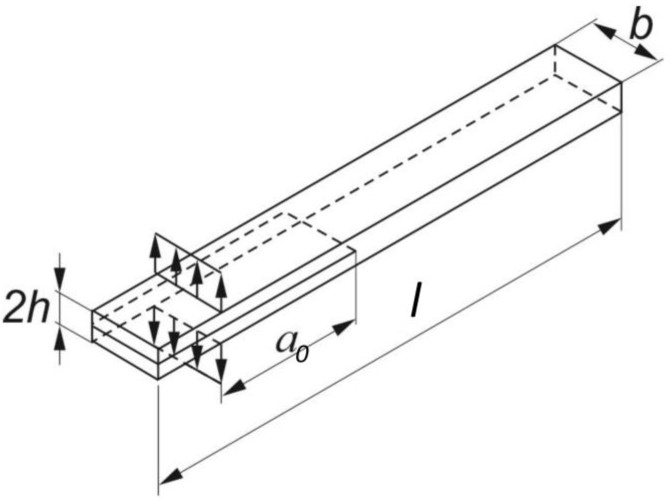
The DCB specimens, according to ASTM D5528.

The DCB specimens for mode I opening were realized according to ASTM D5528, as shown in Figure 4, using organosheets produced by Bond Laminates.

Five specimens were produced 4 mm thick, 25 mm large, and 200 mm long using plain weave laminate cutouts. Five more specimens of the same size were produced using cutouts from a twill weave laminate. These dimensions are, respectively, h, b, and l in Figure 4. The material used is Tepex Dynalite 104-RG601(4) for the plain weave laminate and 104-RG601(8) for the twill weave laminate. Before testing, the crack was carefully propagated for an additional 30 mm along the edge to have an initial crack 50 mm long. The organosheets used for the base of T-Joint specimens were considered orthotropic homogeneous. The elastic modulus, shear modulus, and Poisson coefficient are reported in Table 1.

**Figure 5:**
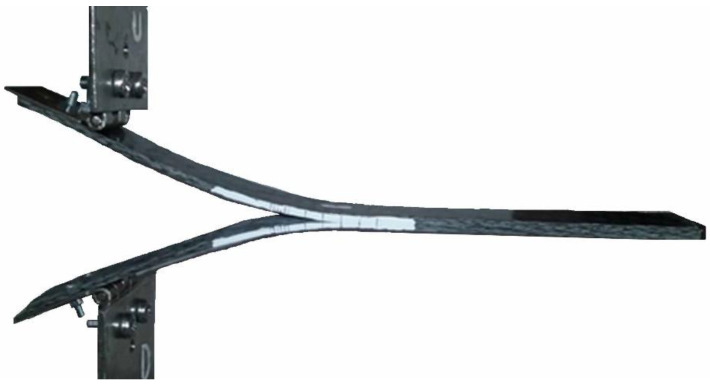
Testing of the DCB specimens.

The tests of the DCB specimens were conducted using the UMT universal machine CETR Multi-Specimen Test System. Figure 5 shows a DCB specimen during the test. The force sensor used has a maximum range of 1000 N. The displacement control was used at a 0.5 mm/min rate for all the tests. The acquisition frequency was set to 5 Hz, given the slowness of the test. The strain energy release rate $G_{I}$ was calculated based on the optical observation in correspondence to the crack initiation and during its propagation at each defined step (five steps of 1 mm and subsequent steps of 5 mm up to a total of 25 mm). The observation of the crack propagation during the experiments was exclusively conducted using a camera, which enabled precise tracking of the crack front’s development along the notches on the DCB specimen’s lateral edge.

### 3.2. Hybrid T-Joint Specimens

**Figure 6:**
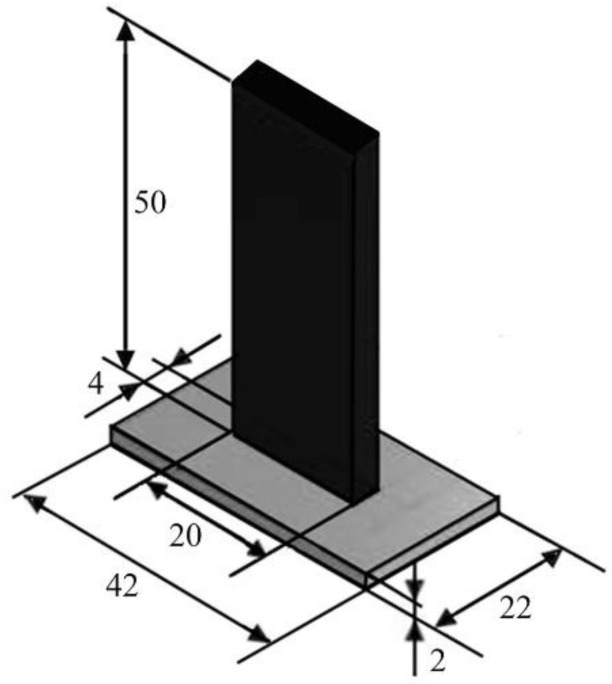
Design of the T-joint specimen. All dimensions are in millimeters.

The hybrid T-joint specimen includes a 2 mm thick composite laminate base and an overmolded stem, which creates an interface area of 4 mm × 20 mm. The geometry, which is shown in Figure 6, tends to emulate a typical structural connection found in various applications such as frame structures, furniture, construction, and especially in aerospace and automotive industries. These joints are commonly used to connect panels or components at right angles, forming a ‘T’ shape, which is integral in creating frames, supports, or reinforcing structures where one part overlaps another at a perpendicular angle.

The base is a rectangular cutout from a laminate made of polypropylene reinforced with 50 wt% glass fibers with balanced woven fabrics: Tepex Dynalite 104-RG600(4), plane weaves manufactured by Bond-Laminates GmbH, Brilon, Germany. The stem is polypropylene reinforced with 30 wt% long glass fibers (Celanese, Celstran PP-GF30-0304, Dallas, TX, USA). The specimens were obtained by injection overmolding of the laminate cutouts placed inside a mold cavity. The material of the overmolded stem was considered elastic and homogeneous isotropic. The elastic modulus and Poisson coefficient are reported in Table 2.

For the overmolding process, the process settings reported in Table 3 were used as they allowed for reaching the highest welding strength.

**Figure 7:**
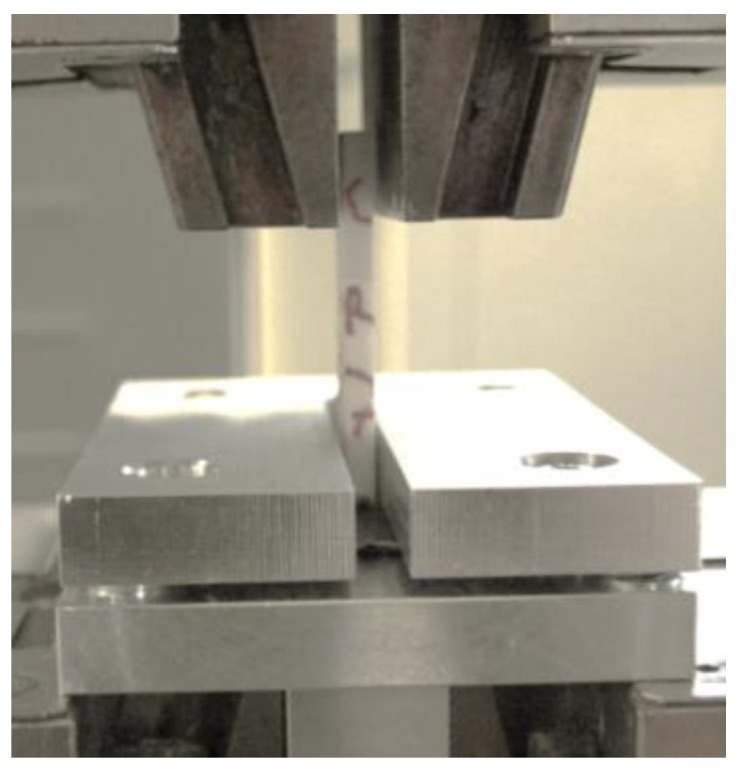
Clamping of the T-joint specimens.

The tensile tests of the T-joint specimens were conducted on a universal tensile testing machine with a load cell of 5 kN. The basis was not clamped but secured using a steel plate with a central rectangular hole with sides 1 mm longer than the sides of the interface area. The clamping of the T-join specimen is shown in Figure 7.

## 4. Modeling

Determining stress and displacement at the crack tip requires a sensitivity analysis of the parameters defining the cohesive zone model to be applied in the simulation that replicates the experimental results. A sensitivity analysis was performed for the DCB and the T-join specimens. The stem of the T-joint specimen was considered non-deformable. This assumption allowed for considering the tensile stress and displacement value obtained from the tensile test plot as a first tentative to represent the cohesive law’s parameters. The geometry of the T-joint specimen allowed for a pure mode I opening and the identification of the values of $T_{n}^{max}$ as the ratio between the load and area of the section.

Both the testing conditions of the DCB and the T-Joint specimens were simulated in Ansys 15.0 Mechanical APDL to evaluate the crack propagation. For the DCB specimen, crack propagation was rigorously modeled using both 2D and 3D finite element analyses. In the 2D model, the crack was represented using INTER202 elements, which implement the cohesive law. The bulk material was discretized using PLANE182 elements. The INTER202 elements were inserted after meshing the base material with PLANE182. Both cohesive and structural elements are 4-node elements with 2 degrees of freedom per node, corresponding to translations in the X and Y directions, selected under a plane strain condition.

The 3D model of the DCB was primarily developed to confirm the correspondence with the results from the 2D plane strain model. For the bulk material, SOLID185 elements were used, while the cohesive layer was modeled with INTER205 elements. Both are 8-node elements with linear shape functions, and each node has 3 degrees of freedom corresponding to translations along the Cartesian axes.

**Figure 8:**
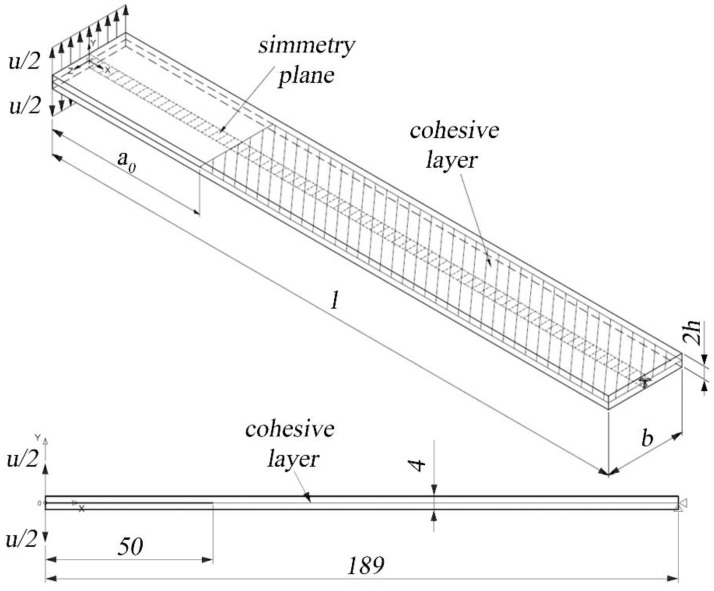
Model of the DCB specimen and boundary conditions for the finite element analysis. The symmetry plane is the XY plane while the cohesive layer lays in the ZX plane. All dimensions are in millimeters.

For the T-Joint specimens, as shown in Figure 8, the laminate and the stem were modeled with Solid185 elements, while the interface was modeled with Inter205 elements. In the cohesive model approach, extreme refinements of the mesh for the checking of singularities are not necessary since the maximum stress is limited by the value $T_{n}^{max}$. Conversely, the mesh should be fine enough to properly describe the crack’s propagation. 

In this study, a mesh convergence study was conducted to ensure the accuracy and reliability of the results obtained from the cohesive zone modeling (CZM) simulations. This study involved systematically refining the mesh near the fracture zone, where stress concentrations were expected to be highest, and the impact on key output variables such as stress intensity factors and crack propagation patterns could be observed.

Initially, a baseline mesh was established, and then it was progressively refined. At each stage of refinement, the simulation was re-run, and the results were compared with those of the previous mesh. Convergence was assumed to have been achieved for the element size equaled to 0.05 mm where the difference for the maximum load was less than 5% when compared to that of the finer mesh. This process ensured that the mesh was sufficiently fine to capture the detailed behavior of the interface fracture without being overly dense, which would unnecessarily increase computational resources and time.

A displacement of 30 mm was applied in 30 sub-steps and split on the two sides of the specimen. The nodes on the opposite sides of the opening cannot translate, and the nodes on the symmetry plane cannot move in the Z-direction.

**Figure 9:**
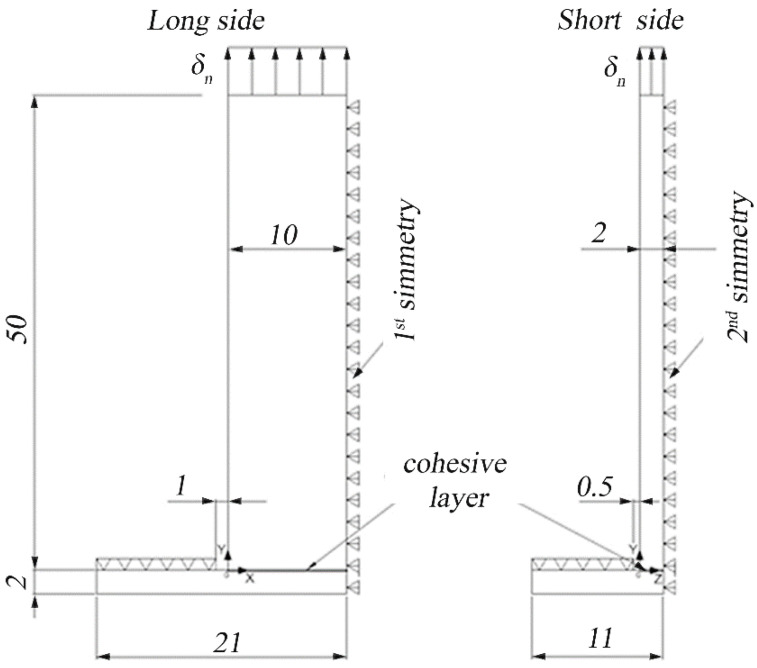
Model of the T-joint specimen and boundary conditions for the finite element analysis. All dimensions are in millimeters.

As shown in Figure 9, a one-fourth symmetry was used for the T-joint specimen. The elements at the interface are 0.25 mm on the long side of the interface and 0.05 mm on the short side to have 40 elements on each side. A displacement of 0.4 mm was applied to the upper face in 60 sub-steps of 0.0065 mm. The elements of the base that are 0.5 mm far from the longer edge and 1 mm far from the shorter edge are constrained in the vertical direction.

## 5. Results and Discussion

### 5.1. DCB Specimens

**Figure 10:**
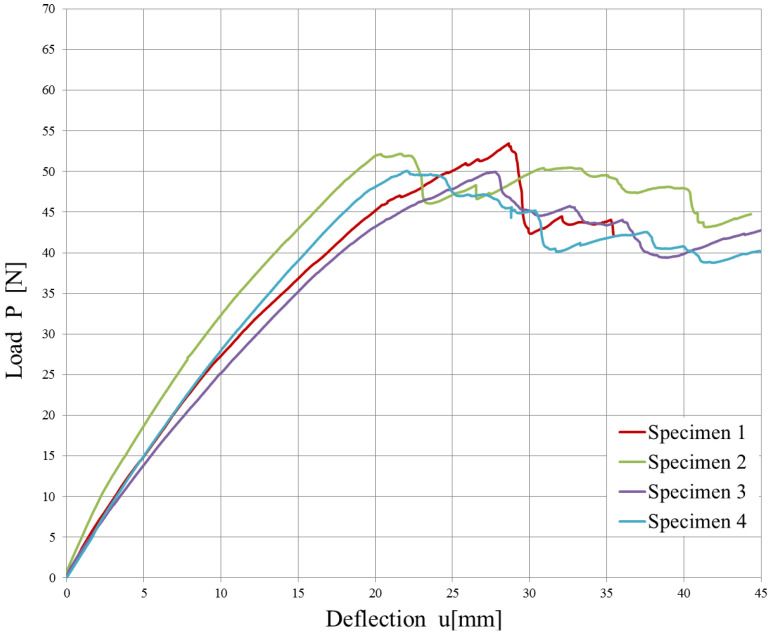
Load vs. displacement plot for 4 DCB specimens cut from the plain weave laminate (failure in acquisition occurred during the test of one of the five specimens).

**Figure 11:**
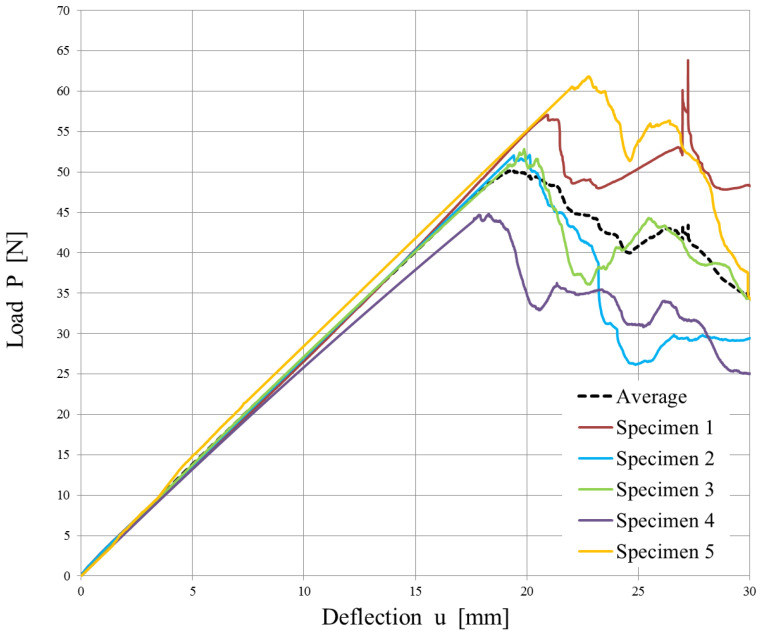
Load vs. displacement plot for the 5 DCB specimens cut from the twill weave laminate.

The experimental data from the opening of the DCB specimens are reported in Figure 10 and Figure 11. A mean value of the Strain Energy Release Rate was calculated using all three methods defined by the ASTM D5523 standard [39] for every millimeter of crack propagation. 

**Figure 12:**
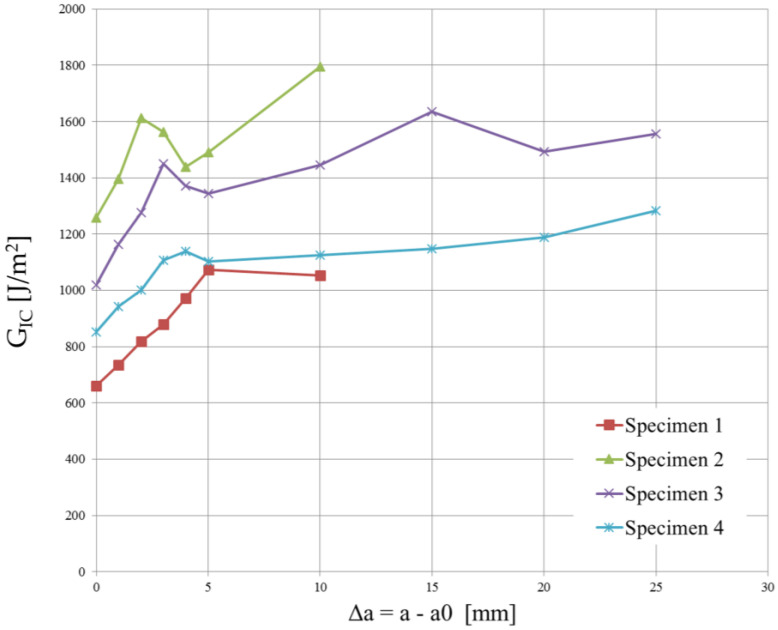
Critical energy release rate vs. delamination length plot for the 4 DCB specimens cut from the plain weave laminate.

**Figure 13:**
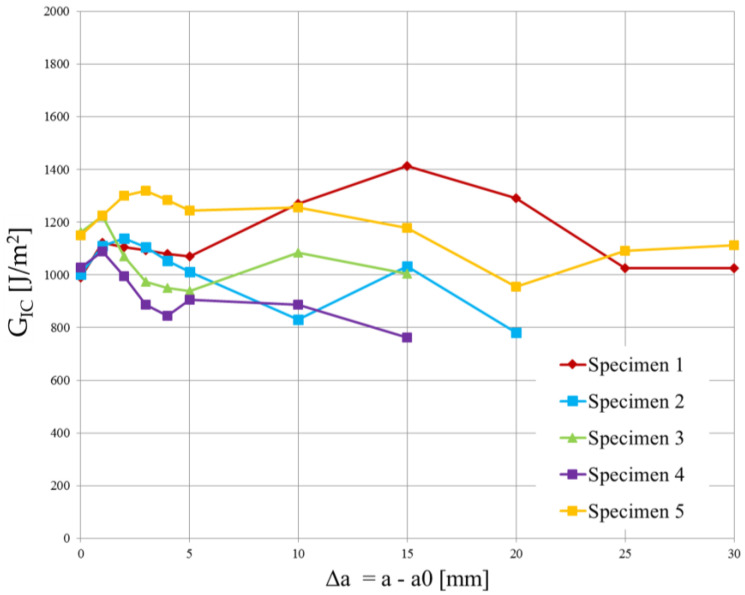
Critical energy release rate vs. delamination length plot for the 5 DCB specimens cut from the twill weave laminate.

Since the strain energy release rate is almost constant, as can be observed in Figure 12 and Figure 13, the overall mean was assumed as the critical energy release rate $G_{IC}$. Its value is reported in Table 4. Although the specimens were manufactured using different laminates, the experimental results led to obtaining very similar values of $G_{IC}$.

The high variability in the measured delamination lengths in Figure 13 can be attributed to a combination of factors related to the material’s weave structure, inhomogeneities, manufacturing inconsistencies, initial flaw characteristics, test conditions, environmental factors, damage mechanisms, and data interpretation and measurement accuracy. 

Even though the T-join specimens were realized using plain weave laminates, the cohesive parameters were determined using the experimental tests conducted on twill specimens because they were produced using a single laminate. Moreover, a lower value of $G_{IC}$ is in favor of safety.

**Figure 14:**
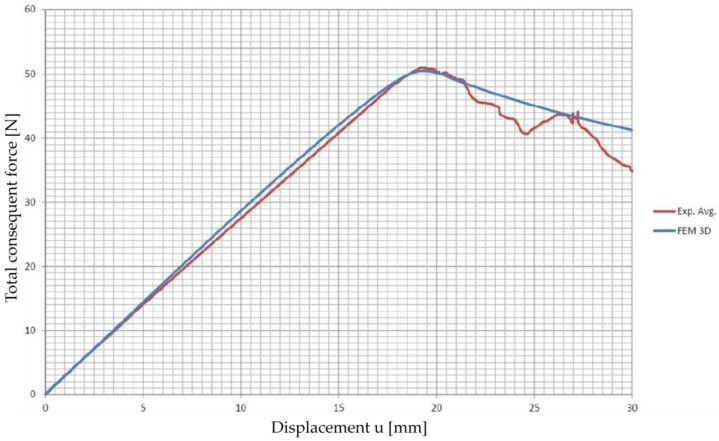
Comparison between the experimental results from the mode I opening test conducted on the DCB specimens and the simulated load vs. displacement plot.

An average experimental curve was calculated and used to calibrate the CZM parameters. Table 5 reports the coefficients of the traction-separation law that defines the behavior of the specimens. A calibration of the cohesive parameters via inverse analysis was undertaken by recalibrating the theoretical model, particularly adjusting the maximum cohesive stress value. This recalibration was informed by a parametric analysis, leading to a maximum tension $T_{n}^{max}\;$= 6.7 MPa. This process involved fine-tuning the model to ensure that the simulated behavior of the DCB specimen’s crack propagation corresponded closely to the observed experimental data. The inverse analysis method effectively allowed for the model to replicate the experimental outcomes, ensuring that the cohesive parameters accurately represented the material’s response. The comparison between the experimental results from the mode I opening test conducted on the DCB specimens and the simulated load vs. displacement plot is shown in Figure 14. The simulation of mode I opening of the DCB specimen, where the interface is modeled with cohesive elements that use a bi-linear law, matched the experimental results.

### 5.2. T-Joint Specimens

**Figure 15:**
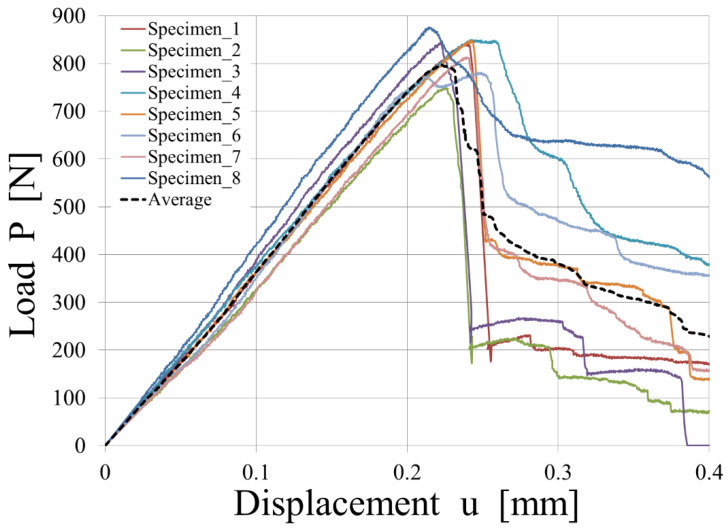
Experimental load vs. displacement plot of the T-joint specimens.

The CZM model was applied to model the interface of the T-joint specimens. In our modeling approach, we concentrated on understanding and predicting mode I fracture behavior in full-thermoplastic hybrid composites. However, it is pertinent to mention that the T-joint specimens, due to their specific geometry and loading conditions, might also experience minor shear effects, characteristic of mode II fracture. This effect was not directly addressed in our study. Future research could benefit from extending the cohesive zone modeling approach to incorporate mode II fracture behavior, thus providing a more comprehensive understanding of the fracture mechanics in such composite structures. The experimental results are shown in Figure 15.

The parameters that describe the CZM model are reported in Table 6. They were determined considering the value of G_IC obtained from the DCB tests.

**Figure 16:**
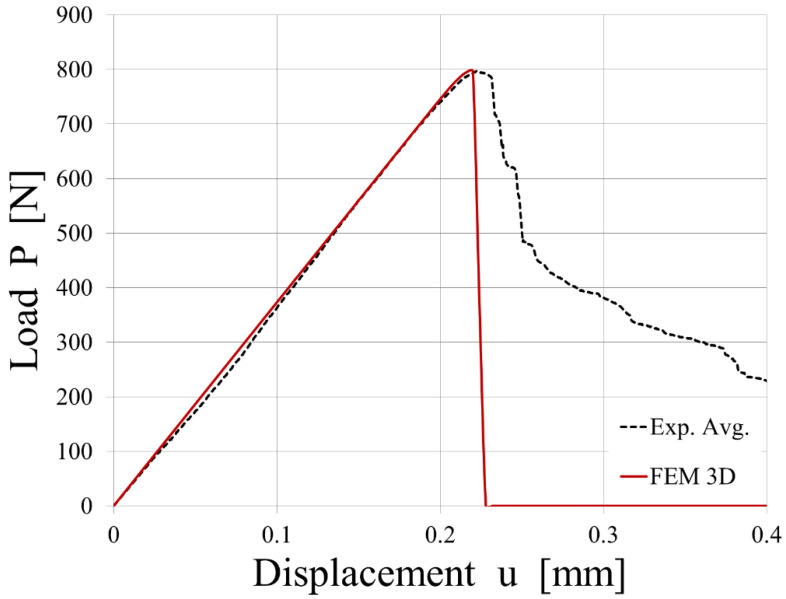
Comparison between the experimental results from the tensile tests conducted on the T-joint specimens and the simulated load vs. displacement plot.

An average experimental curve was calculated and used to calibrate the CZM parameters. The comparison between the experimental results from the tensile tests conducted on the T-joint specimens and the simulated load vs. displacement plot is shown in Figure 16.

The simulation of the tensile tests conducted on the T-joint specimens, where the interface is modeled with cohesive elements that use a bi-linear law, matched the experimental results in the linear-elastic field. On the contrary, the simulation predicts a very brittle breakage, whereas the experimental results, represented by an average value, show a progressive one. 

The differences in Figure 16 between the experimental and simulated results can be attributed to factors like the overmolding technique, thermal gradient effects, interface characterization, simulation model accuracy, and the influence of process parameters on bond strength. According to Jiang et al. [40], hybrid fiber-reinforced thermoplastic composites fabricated by the overmolding technique offer significant mechanical performance due to the combination of continuous and short-fiber-reinforced thermoplastics. However, they also note that the heterogenous interface between these two materials, generated during overmolding, can have varying adhesion qualities due to unmatched thermal properties, affecting the interfacial properties. They also discuss how the thermal gradient affects interfacial properties in hybrid structures, showing that a decreased thermal gradient can greatly improve interfacial shear strength due to the higher crystallinity and larger spherulite size of the polypropylene matrix at the interface. This finding suggests that thermal gradients during manufacturing could significantly influence the discrepancies observed in Figure 16.

Looking in detail at the experimental results, samples 1, 2, and 3 show brittle behavior matched by simulation data, whereas 4, 5, 6, 7, and 8 show a more progressive breakage. In those latter samples, the phenomenon of fiber-bridging was observed. 

The experimental results from each test show extended variability from one specimen to the other, and at least two different main behaviors can be identified. When delamination occurs without secondary effects, a bi-linear law predicts well the experimental behavior. When delamination shows a slower propagation due to fiber-bridging, the tri-linear law could offer a better prediction.

## 6. Conclusions

This study provided a comprehensive analysis of the fracture behavior in full-thermoplastic hybrid composites, utilizing a cohesive zone modeling (CZM) approach. The key conclusions drawn from our investigation are as follows:

- The CZM approach effectively modeled the fracture strength of full-thermoplastic hybrid composites made by forming and overmolding organosheets. This methodology was particularly adept at replicating the linear-elastic behavior of T-joint specimens under tensile testing.
- The experimental results highlighted the occurrence of fiber-bridging during delamination. This phenomenon was successfully replicated in the simulations, illustrating the CZM’s ability to capture complex behaviors within the composite interface.
- While the model predictions aligned with experimental observations in the linear-elastic domain, discrepancies emerged in post-yield behavior. The simulations predicted a more brittle fracture compared to the progressive breakage observed in experiments, suggesting areas for future refinement in the modeling approach.
- The study established a direct correlation between the mode I fracture toughness of DCB specimens and the behavior of T-joint specimens. This finding underscores the importance of fracture toughness in predicting the performance of complex joint configurations in composite materials.
- Our findings have significant implications for the manufacturing of full-thermoplastic hybrid composites. Understanding the fracture behavior and interface characteristics can guide design decisions and optimize manufacturing processes for enhanced performance.

While this study provides valuable insights into the fracture behavior of full-thermoplastic hybrid composites, its methodology has limitations in terms of the scope of modeling, the novel application of CZM, the ability to capture complex material behavior, and the generalizability of its findings. These limitations should be considered when interpreting the study’s results or applying its insights to other scenarios in the field of composite materials. Future work should focus on refining the CZM parameters to better capture the post-yield behavior and progressive fracture mechanisms. Additionally, expanding the study to include other types of fractures and material combinations would broaden the applicability of the research findings.

In summary, this study has advanced our understanding of the fracture behavior in full-thermoplastic hybrid composites, demonstrating the applicability and limitations of CZM in this context. The insights gained from this work will pave the way for the more accurate modeling and improved design of composite structures.

## Figures and Tables

**Table 1:** Elastic orthotropic material properties of the organosheet.

Elastic Modulus [Gpa]	Shear Modulus [Gpa]	Poisson Coefficients
E_x_ = 17.5	G_xy_ = 2	ν_xy_ = 0.4
E_y_ = 2.5	G_xz_ = 1.5	ν_xz_ = 0.25
E_z_ = 17.5	G_yz_ = 2	ν_yz_ = 0.05

**Table 2:** Elastic homogeneous isotropic material properties of the stem.

Elastic Modulus [Gpa]	Poisson Coefficients
E = 4.2	ν = 0.4

**Table 3:** Process settings used for manufacturing the T-joint specimens.

Melt Temperature [°C]	Packing Pressure [bar]	Mold Temperature [°C]	Laminate Temperature [°C]
260	200	80	150

**Table 4:** Average values and standard deviation of $G_{IC}$.

DCB Specimen	$G_{IC}$ [J/m^2^]	
	Mean	St. Dev.
2 × 104-RG601(4) plain weave	1214	280
1 × 104-RG601(8) twill weave	1062	105

**Table 5:** Model parameters used for the CZM simulation of the T-joint specimens.

$G_{IC}$ [J/m^2^]	$T_{n}^{max}$ [MPa]	$\delta_{n}^{*}$ [mm]	α
1062	6.7	0.314	0.1

**Table 6:** Traction-separation coefficients and strain energy release rate.

$G_{IC}$ [J/m^2^]	$T_{n}^{max}$ [MPa]	$\delta_{n}^{*}$ [mm]	α
1062	10.3	0.206	0.36

## Data Availability

The data that support the findings of this study are available from the corresponding author, G.L., upon reasonable request.
